# Fluid evolution and related fluid–rock interactions of the Oligocene Zhuhai sandstones in the Baiyun Sag, northern margin of the South China Sea

**DOI:** 10.1038/s41598-023-41428-3

**Published:** 2023-08-28

**Authors:** Bing Tian, Yihan Yuan, Jun Tang, Shanshan Zuo, Youwei Zheng, Ming Liu, Cheng Guo

**Affiliations:** 1 Institute of Mining and Coal, Inner Mongolia University of Science and Technology, Baotou, 014010 China; 2 Institute of Science, Inner Mongolia University of Science and Technology, Baotou, 014010 China

**Keywords:** Ocean sciences, Solid Earth sciences, Energy science and technology

## Abstract

Pore fluids control the diagenetic processes and storage spaces of deep clastic rock reservoirs and have become a major area of interest within the fields of sedimentology and petroleum geology. This paper aims to relate the diagenetic processes of the Oligocene Zhuhai sandstones in the Baiyun Sag to pore fluids varying with burial depth. The types and distribution patterns of authigenic minerals are investigated through analysis of petrographic, mineralogical, and geochemical features to illustrate the origin and flow patterns of pore fluids and their influences on reservoir diagenesis. Strong cementation of eogenetic carbonate cement near the sandstone–mudstone interface was a consequence of material migration from adjacent mudstones on a large scale. The pore fluids were mainly affected by microbial methanogenesis and carbonate mineral dissolution in adjacent mudstones during eogenesis. The pore fluids were diffusively transported in a relatively open geochemical system within a local range. Support for this model is provided by the heavier stable isotopic values present in eogenetic calcite and dolomite. Feldspar dissolution during early mesogenesis was spatially accompanied by the precipitation of authigenic quartz and ferroan carbonate cement. Pore fluids in this period were rich in organic acids and CO2, and their migration mechanism was diffusive transport. The obviously lighter carbon and oxygen isotopic compositions of the ferroan calcite support this inference. During late mesogenesis, the input of deep hydrothermal fluid might have been partly responsible for the precipitation of ankerite, barite and authigenic albite. Oil charging may have inhibited carbonate cementation and compaction, accordingly preserving porosity, and together with authigenic kaolinite, might have promoted the transition of the reservoir from water wet to oil wet to the benefit of oil entrapment. The findings reported here shed new light on the evaluation and prediction of sandstone reservoirs that have experienced multiple periods of fluid flow.

## Introduction

Pore fluids are nearly ubiquitous in clastic rocks and, with increasing burial depth, exert a crucial influence on the petrophysical properties through various fluid–rock interactions^1–3^. Aggressive pore fluids strongly corrode aluminium silicate minerals and carbonate minerals in deep clastic rock reservoirs, creating (or redistributing) secondary pores of a certain scale, thereby significantly (or slightly) improving reservoir porosities. The concomitant precipitation of secondary minerals, mainly in the form of pore-filling minerals, due to mass transfer by pore-fluid flow plays a negative role in reservoir permeability^3–7^. Identifying the origin and flow patterns of pore fluids is crucial for research on sandstone–shale diagenesis and storage properties^8^. Complex sandstone reservoirs interbedded with mudstone can be complicated by the potential for multiple stages of evolving pore fluids and corresponding fluid–rock interactions during progressive burial. To define and prioritize reservoir targets, the sources, flow patterns and spatiotemporal distribution of pore fluids must be understood.

Stable isotope ratios are commonly employed to constrain (1) the sources of pore fluids, (2) the pathways and timing of fluid events, (3) the formation temperatures of multiple-stage cements, and (4) the material sources of diagenetic byproducts^9–13^. The stable isotopic compositions of carbon and oxygen are highly stable in different fluid systems that have deep circulation characteristics. The degree of oxygen isotope fractionation between fluids and minerals is reduced with increasing formation temperature (surface to ~ 300°C^14^). The δ^18^O value preserved in cement can serve as a proxy record of cementation temperature. Thus, it is a useful indicator to infer the time of cement formation and to clarify the evolution of pore fluids when given a reasonable pore-fluid δ^18^O value^15,16^. Compared to the δ^13^C value in the original carbon pool, that preserved in cement is heavier by approximately 9–10 ‰ due to carbon isotope fractionation. Thus, δ^13^C values can be used to trace the external or internal sources of carbon^12,13^ and to address frequently asked questions related to fluid–rock interaction^16–20^. On the basis of these two stable isotope systems, combined with the regional evolution history, the physicochemical and flow features of fluids throughout the whole diagenetic process can be reconstructed^21,22^.

The Oligocene Zhuhai Formation in the Baiyun Sag, northern margin of the South China Sea, is a vital target of offshore petroleum exploration and development in China^23^. The interval consists of multistage, shelf-margin deltaic fine- to medium-grained siliciclastics. Pore fluids related to the interbedded mudstones are likely to have transported large amounts of ions driven by various mechanisms with increasing burial depth, which may have exerted a critical influence on the diagenetic processes of the sandstones^24–26^. Therefore, the Zhuhai Formation is an ideal archive for research on the characteristics of fluid evolution and related fluid–rock interactions. In this study, we investigated the type, nature and distribution patterns of diagenetic minerals in the Zhuhai sandstone reservoirs in the Baiyun Sag and clarified the evolution of the pore fluids responsible for those diagenetic processes.

## Geological setting

**Figure 1:**
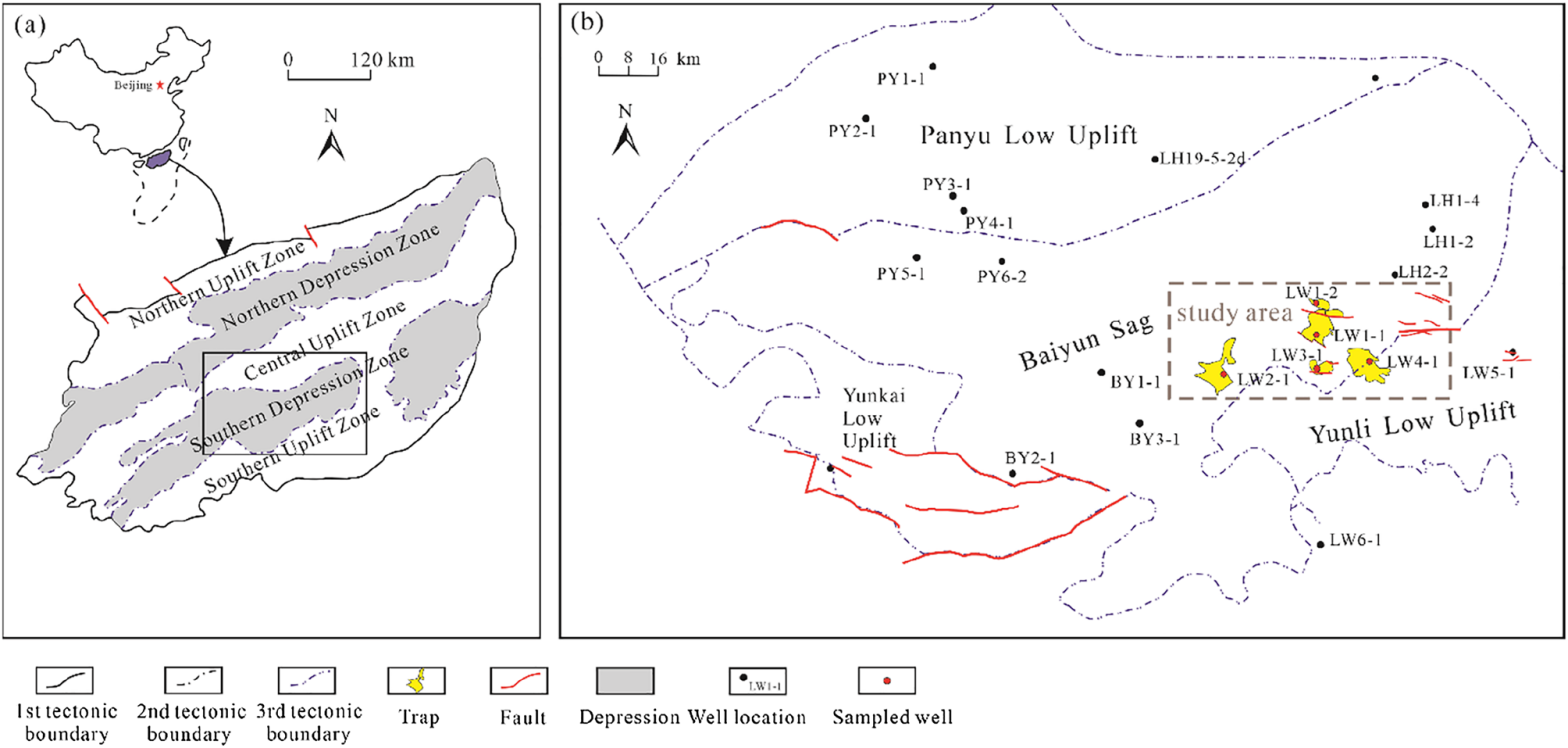
(**a**) Tectonic unit divisions of the Pearl River Mouth Basin, South China Sea. (**b**) Structural map and well locations of the Baiyun Sag (modified from ^26^ by using CorelDRAW Graphics Suite 2018 v20.0.0.633 https://www.corel.com/cn).

**Figure 2:**
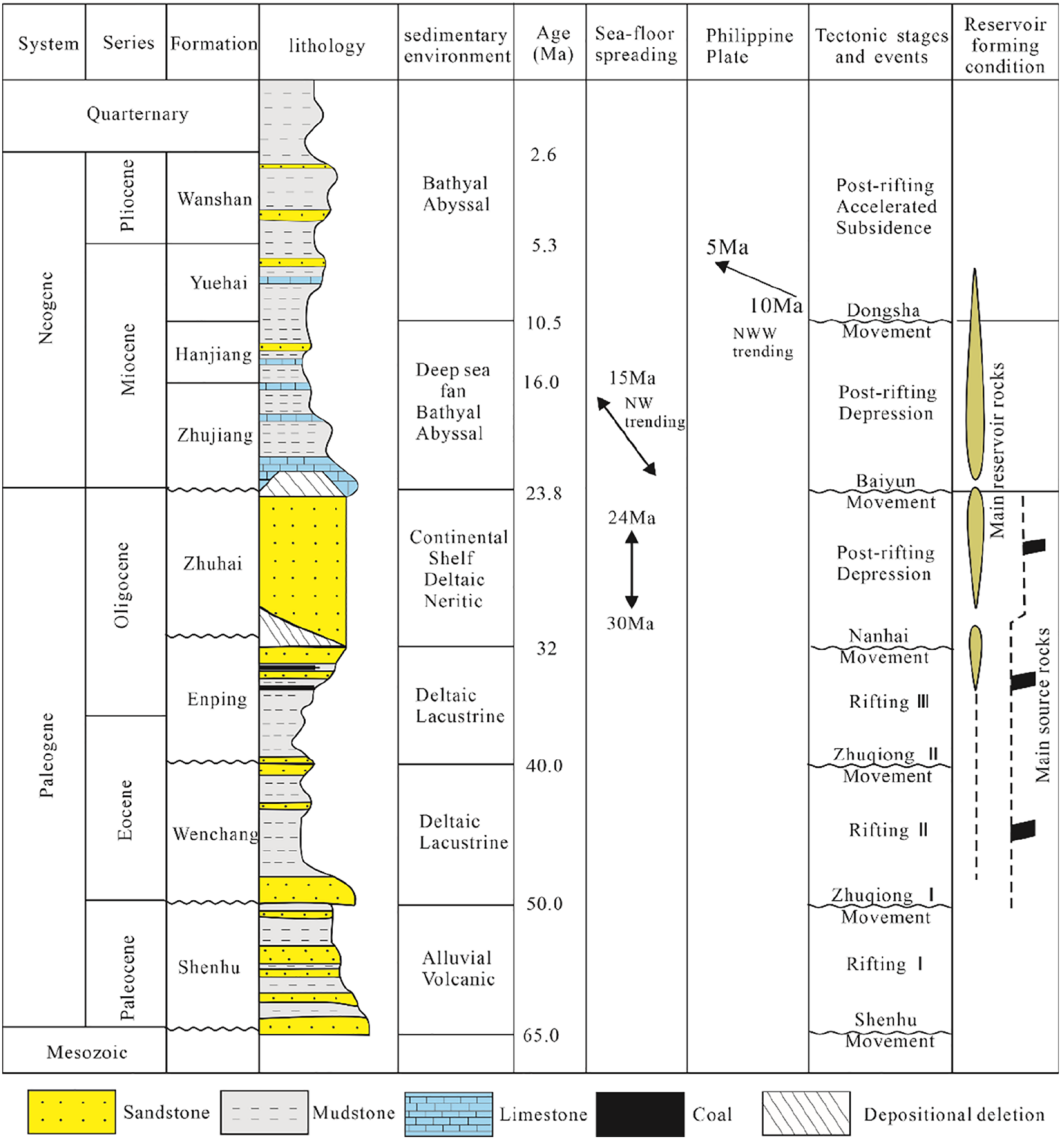
Schematic Cenozoic stratigraphy of the Baiyun Sag with tectonic evolution stages and the major reservoir formation elements (modified from ^26^ by using CorelDRAW Graphics Suite 2018 v20.0.0.633 https://www.corel.com/cn).

The Baiyun Sag is located on the northern margin of the South China Sea and covers an area of 1.2 × 10^4^ km^2^ with water depths of 200 m to 2000 m (Fig. 1a). As a Cenozoic extensional basin, the sag can be divided into a synrift stage between the Eocene and early Oligocene (65–32 Ma) and a postrift stage between the late Oligocene and the present (32–0 Ma) (Fig. 2)^27^. The study area, as one of the most hydrocarbon-rich deep-water areas, lies in the southeastern Baiyun Sag (Fig. 1b).

The sag is filled with Cenozoic sediments and, in ascending order, consists of the Palaeogene Shenhu, Wenchang, Enping and Zhuhai Formations; the Neogene Zhujiang, Hanjiang, and Yuehai Formations; and Quaternary groups (Fig. 2). The evolution of the synrift stage was mainly affected by the Zhuqiong movement and the Nanhai movement and can be subdivided into three stages. The studied section, i.e., the Oligocene Zhuhai Formation, was deposited in the late synrift stage and is mainly composed of Palaeo-Pearl River shelf-margin deltaic sandstones^23^.

The tectonic activity was relatively quiescent during the Zhuhai depositional stage. Shelf breaks stably developed in the southern deep-water area of the sag. Fluctuations in sea level and salinity were frequent and rapid. The Baiyun Sag was characterized by a wide distribution of oscillatory continental–marine transition sedimentary environments. Large-scale grey‒greyish white sandstones interbedded with brown–dark grey mudstones developed in the sag, forming a laterally extensive Palaeo-Pearl River shelf-margin delta system. The sedimentary facies were dominated by subaqueous distributary channels, mouth bars and sheet sands in the southern deep-water area of the sag and inter fingered with more subaqueous inter distributary bay and pro delta argillaceous deposits. The thick, organic-rich mudstones are important potential hydrocarbon source rocks for the intercalated thin sandstone reservoirs^27^.

## Samples and methods

Sixty-five core samples with burial depths of 800–3000 m were collected from five wells (Fig. 1). All samples were obtained from delta deposits with various thicknesses. To analyse the underlying correlation between reservoir quality and distance from the sandstone–mudstone interface, samples were selected from different positions within one bed. A total of 210 core analysis data points of Zhuhai sandstones were taken to evaluate porosity and permeability.

Point counting was performed on 116 thin sections of Zhuhai sandstone in the southeastern Baiyun Sag in this study to estimate the modal composition. Thin sections were impregnated with blue epoxy under vacuum to identify visual porosity and stained with potassium ferricyanide and Alizarin Red S to aid in distinguishing carbonate. A total of 300 points were counted for each thin Sect.^28^. For the accurate estimation of cement and pore contents, 20 micrographs per thin section were first obtained under a Zeiss microscope, and the targets per micrograph were then sketched and calculated using the AxioVision software Rel and Image-Pro Plus software. Finally, the average values of each target area in all micrographs were obtained, which can be regarded as the contents of pores and cement.

All 65 samples were prepared for mineralogical X-ray diffraction (XRD) analysis using an Ultima IV X-ray diffractometer. Samples were X-rayed, centrifuged, glycolated, and heated to 550 °C. XRD analysis was based on the procedure used by^29^ within an error range of 10%. After detailed petrographic analysis, seven representative samples were observed using cathodoluminescence microscopy (CL) to distinguish multiphase carbonate cement. For the spatial morphology of authigenic mineral identification, 22 gold-coated samples were observed under a ZEISS EVO LS15 scanning electron microscope (SEM) equipped with an energy-dispersive spectroscopy X-ray microanalyzer.

Thirty organic matter-free sandstone samples were prepared based on petrological studies for carbon and oxygen isotope measurements. To target specific carbonate cement generations, microsamples (0.35–0.45 mg) of different types of cement were drilled from thick petrographic sections using a microscope-mounted dental drill. Each sample was reacted with 100% ortho-phosphoric acid at 70 °C for 4 to 8 h. The carbon and oxygen stable isotope values were obtained from the CO_2_ liberated from the carbonate cement samples using a Thermo-Finnigan MAT 253 IRMS. The measurement precision was ± 0.014‰ for oxygen and ± 0.02‰ for carbon. The stable isotope data were reported in δ notation relative to the Pee Dee belemnite (V-PDB). δ^18^O_VPDB_ values were converted to δ^18^O_VSMOW_ (Vienna standard mean ocean water) values using the equation δ^18^O_VSMOW_ = 1.03091 × δ^18^O_VPDB_ + 30.91^30^.

Seven core samples were prepared as doubly polished fluid inclusion wafers for microthermometric measurement. Microthermometry was conducted using a calibrated LINKAM THMSG600 stage. The homogenization temperature (Th) was obtained by cycling. Th values were measured using a heating rate of 10 °C/min when the temperature was lower than 80 °C and a rate of 5 °C/min when the temperature exceeded 80 °C. The measured temperature precision for Th was ± 1 °C. All the analyses mentioned above were performed in the Key Laboratory of Petroleum Resources Research, CAS (Lanzhou).

## Results

### Detrital petrology

**Figure 3:**
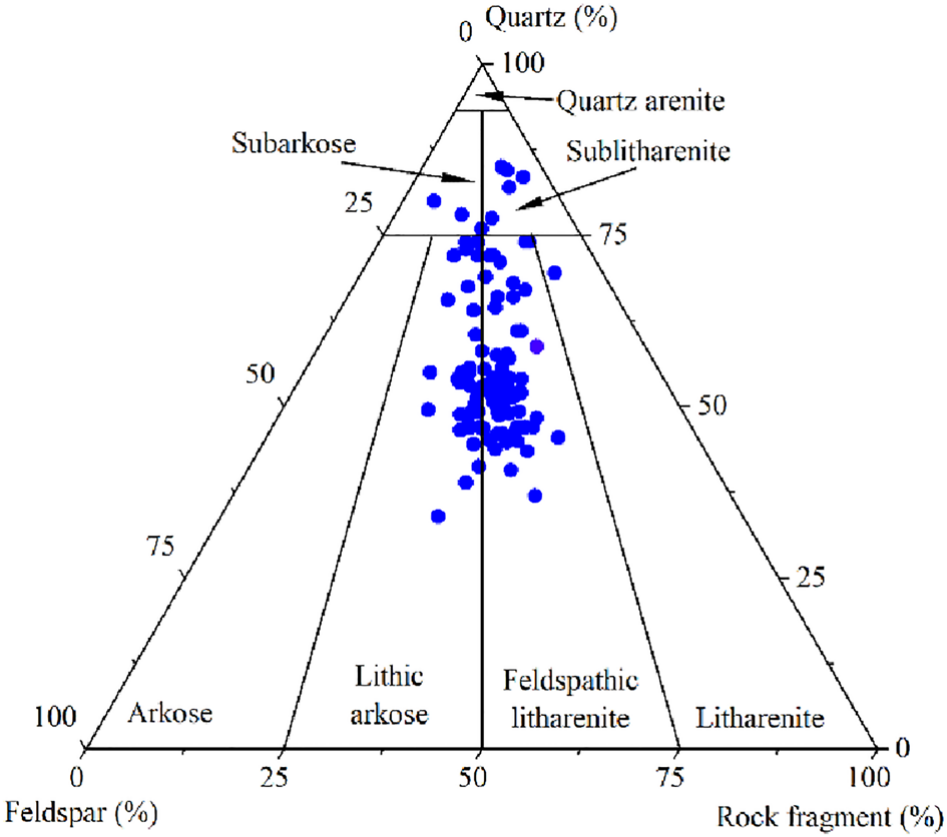
Triangular diagram of the rock composition of the Zhuhai sandstones in the Baiyun Sag. All data come from optical point counting of thin sections.

Compositionally, the studied Zhuhai sandstones are dominantly feldspathic litharenites followed by lithic arkoses (Fig. 3). They are mostly fine- to medium-grained, moderately to well sorted and subangular to subrounded. Based on point-count data, detrital quartz accounts for 34–85% (average 56.5%) and is the predominant framework grain. Detrital feldspar accounts for 3–38.5% (average 20.3%), and K-feldspar (average 14.9%) is more abundant than plagioclase (average 5.4%). The rock fragment content is approximately 4–38.2% (average 23.2%) and consists of 2.5–28% volcanic rocks (average 13.8%), 0.3–26.5% sedimentary rocks (average 2.78%), and 0.3–14.7% metamorphic rocks (average 6.62%). Only small quantities of micas and heavy minerals can be found. The mud content is 0.3–45% (average 4.68%), and the cement content is 0.2–32.3% (average 7.24%). The compositional maturity varies between 0.52 and 5.67, with an average of 1.55.

### Feldspar dissolution

**Figure 4:**
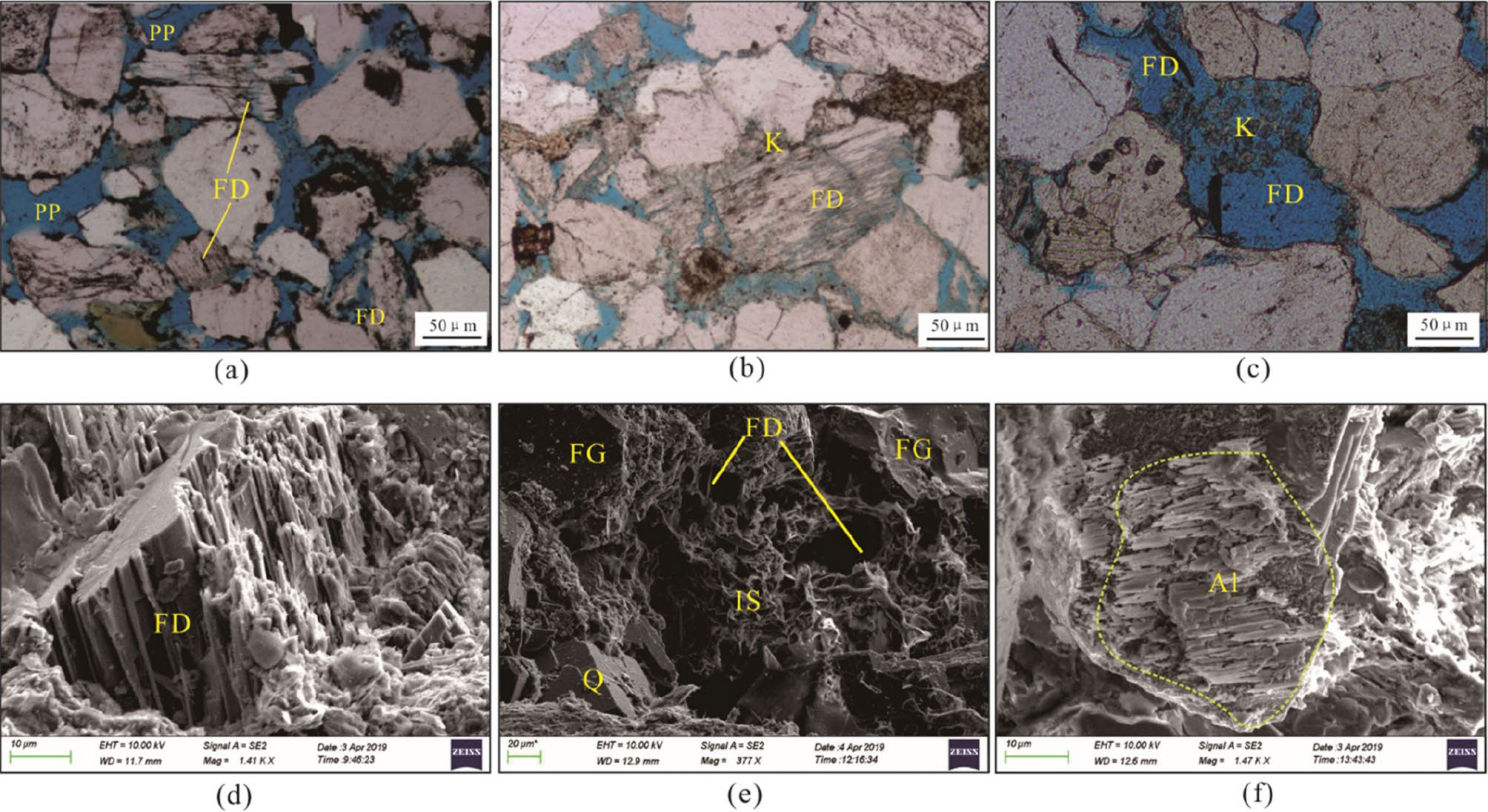
Photomicrographs of feldspar dissolution and associated authigenic minerals in the Zhuhai sandstones. (**a**) LW1-1, 1707.5 m: dissolution of feldspar along the cleavages; (**b**) LW4-1, 1519.8 m: leached feldspar with the precipitation of secondary kaolinite and associated secondary minerals; (**c**) LW1-2, 2429.5 m: feldspar-dissolution mouldic pores and kaolinite precipitation; (**d**) LW1-1, 1707.5 m: SEM micrograph showing leached feldspar along the cleavages; (**e**) LW3-1, 1861.5 m: SEM micrograph showing a feldspar-dissolution pore filled by clay precipitation; (**f**) LW1-2, 2509.5 m: precipitation of albite on leached feldspar. Note: Q = quartz, FD = feldspar dissolution, PP = primary pore, IS = illite and smectite mixed layers, K = kaolinite, and Al = albite.

**Figure 5:**
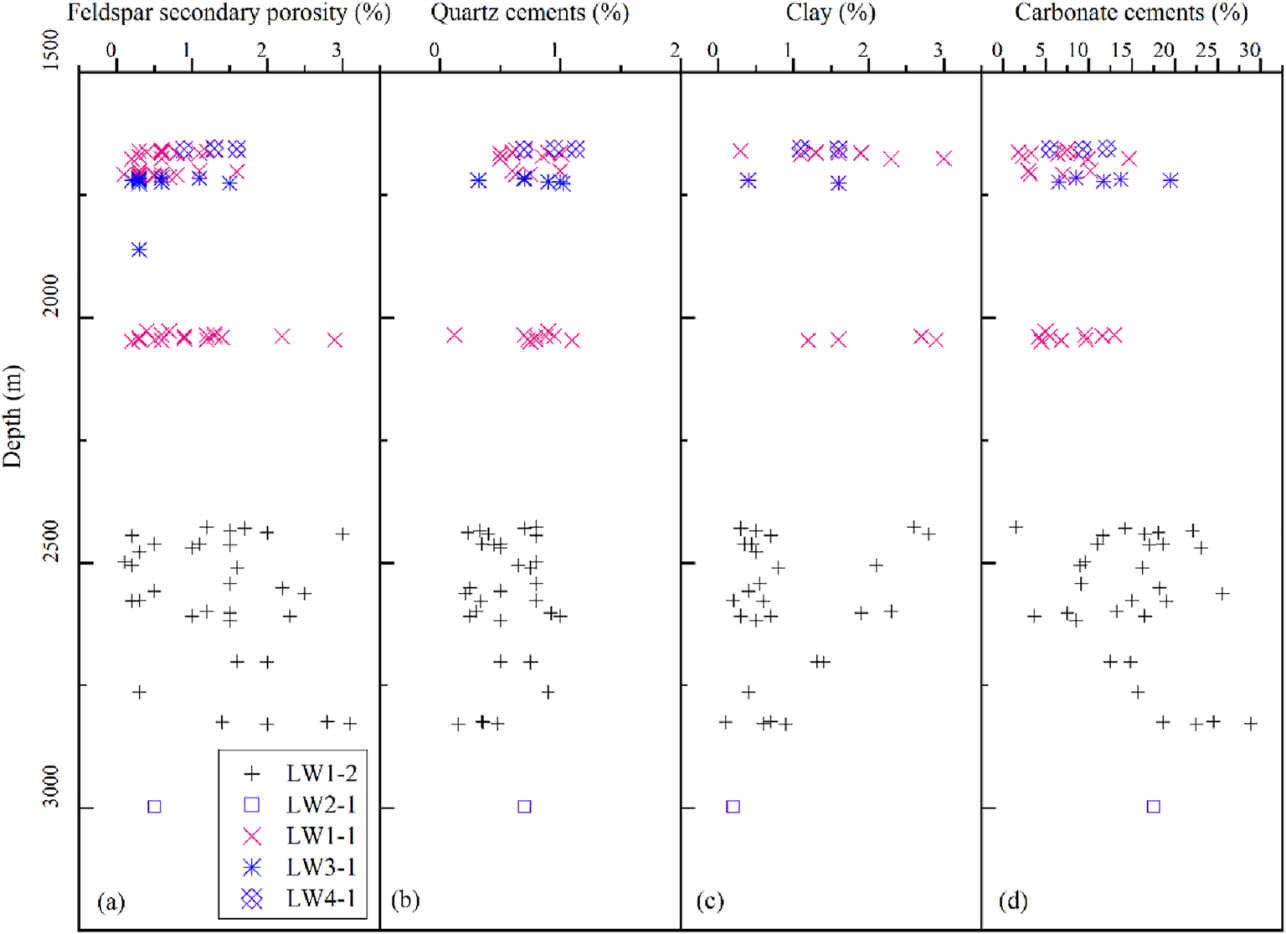
Vertical distribution characteristics of the main diagenetic products in the Zhuhai sandstone. Point-count data of thin sections are used.

**Figure 6:**
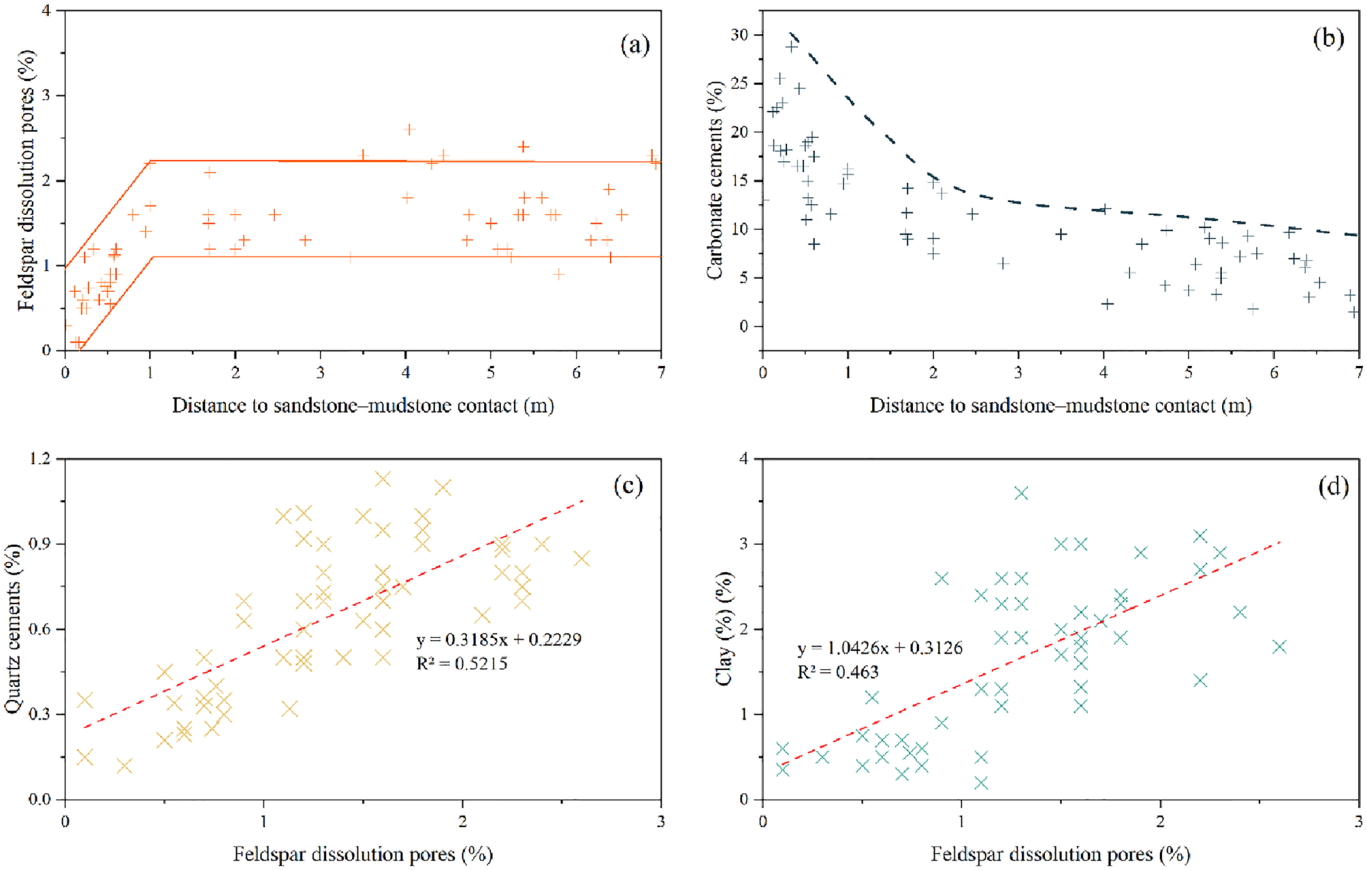
The relationship between the abundance of the main diagenetic products in the sandstones and the distance to the sandstone–mudstone interface. Point-count data of thin sections are used.

Partial to complete feldspar grain dissolution is very common in the Zhuhai sandstones, and most dissolved grains are K-feldspar and locally plagioclase. Feldspar grains preferentially dissolved along the cleavage, revealed as irregular dissolution edges and feldspar residue (Fig. 4a,b,d). Some feldspar grains were almost totally dissolved, forming mouldic pores (Fig. 4c). Spatially, feldspar-dissolution pores are accompanied by pore-filling authigenic clays, albite and quartz cements (Fig. 4c and e). The authigenic albite presents as columnar aggregates of euhedral crystals, and the elongated crystals are parallel to the cleavage of the dissolved K-feldspar (Fig. 4f). The content of dissolved feldspar is 0.1–3.1%, with an average of 1.26%. The quantity of dissolution pores marginally increases with burial depth (Fig. 5a); moreover, there is a marked increase in sandstones more than 1 m away from the sandstone–mudstone contact within a bed (Fig. 6a).

### Carbonate cements

**Figure 7:**
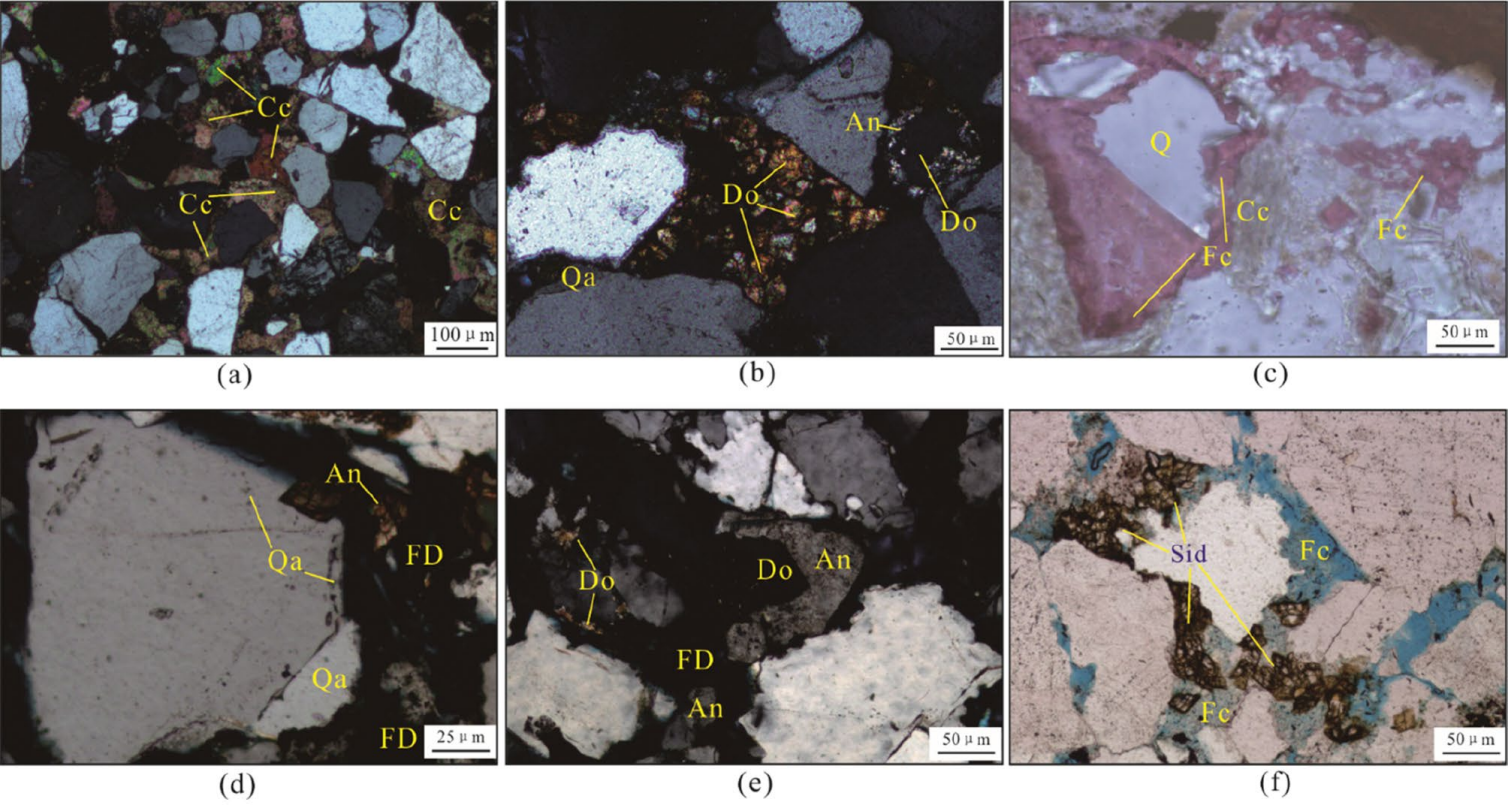
Photomicrographs of carbonate cements in the Zhuhai sandstones. (**a**) LW1-1, 2027.25 m: poikilotopic, blocky calcite filling most primary pores; (**b**) LW1-1, 2037.5 m: dolomite filling intergranular primary pores with zoning and engulfed by ankerite; (**c**) LW1-2, 2563.5 m: calcite replaced by ferrocalcite; (**d**) LW1-2, 2563.5 m: quartz overgrowths replaced by ankerite; (**e**) LW1-1, 2037.5 m: dolomite engulfed by ankerite; (**f**) LW1-2, 2429.5 m: siderite precipitated around detrital grains. Note: Cc = Calcite, Do = Dolomite, Fc = Ferroan calcite, An = Ankerite, FD = Feldspar dissolution pore, Qa = Quartz overgrowth, Sid = Siderite.

Carbonate cements are the most abundant of the secondary minerals, and the mean content is 11.6% (range 0.2–28%) (Fig. 5d). These cements are mainly composed of calcite (Fig. 7a), dolomite (Fig. 7b), ferrocalcite (Fig. 7c), ankerite (Fig. 7d, and e) and siderite (Fig. 7f). Calcite (average 2.54%) is mainly present as poikilotopic blocky or pore-filling crystals with crystal sizes in the range of 5–300 μm, which either infilled primary pores or replaced detrital grains partially or completely. Calcite was observed to be zoned and engulfed by ferroan calcite (Fig. 7c).

Dolomite (average 1.07%) is commonly composed of microsparry or micritic aggregates (5–250 μm) that generally appear as rhombohedral crystals (Fig. 7b and e). Dolomite cements were also observed to fill primary pores between uncompacted framework grains as pore-filling poikilotopic cement.

Ferroan calcite (average 1.69%) is mainly present as isolated crystals filling pores and secondarily scattered euhedral crystals (5–200 μm; Fig. 7c), while ankerite (average 4.62%) mainly occurs as euhedral rhombs (5–150 μm; Fig. 7d) and mosaic clusters (10–250 μm; Fig. 7e). These cements partly occupy fractures and feldspar-dissolution pores and have replaced clastic grains or early carbonate cements (Fig. 7b,c,e). The siderite ranges in colour from light to dark brown and appears as irregular rhombs filling intergranular pores (Fig. 7f).

The carbonate cement content is commonly observed to be higher in sandstones less than 1.0 m away from the sandstone–mudstone interface (Fig. 6b), forming sandstones tightly cemented by calcite and dolomite filling all the intergranular pores (Fig. 7).

**Figure 8:**
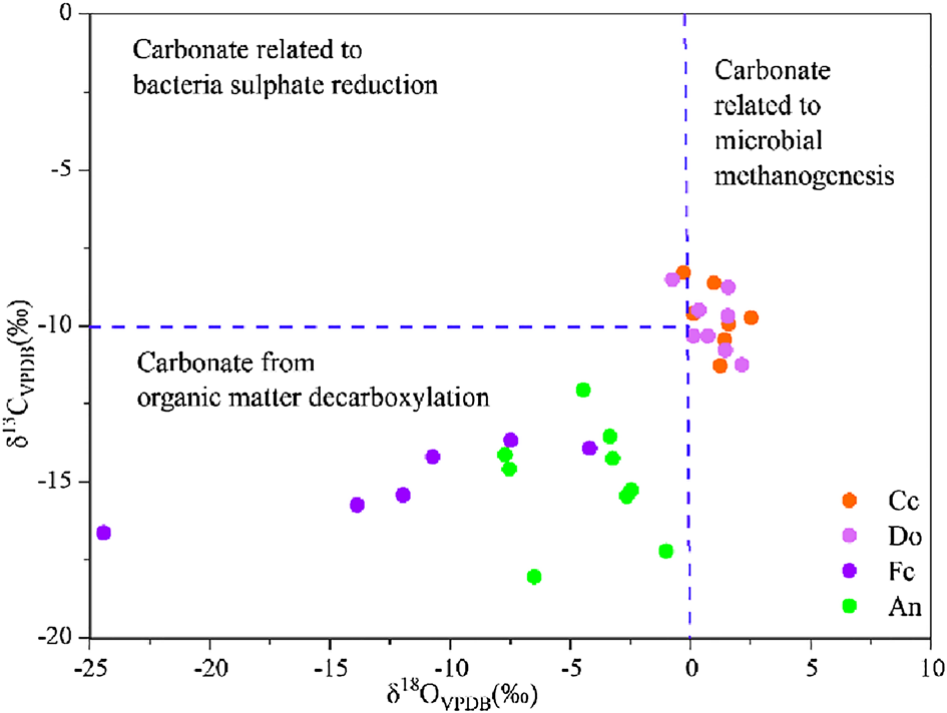
Crossplot of δ^13^C and δ^18^O compositions of carbonate cements in the Zhuhai sandstones. Standard isotopic zones for carbon sources of variable carbonate cements are according to ^32^.

On the basis of the isotopic analysis, the calcite cements have δ^13^C values between − 0.3 and + 2.51‰, and δ^18^O values range between − 11.27 and − 8.28‰. The dolomite cements have a range of δ^13^C values (− 0.76‰ to + 2.12‰) and δ^18^O values (− 11.24‰ to − 8.49‰) that are similar to those of calcite. In contrast, the ferroan calcite cements have significantly lighter δ^13^C values (− 24.42‰ to − 4.19‰) and slightly lighter δ^18^O values (− 16.63‰ to − 13.67‰) (Table 1). For the ankerite cements, the δ^18^O values do not vary much, ranging between − 18.05 and − 12.06‰, but the δ^13^C values are significantly heavier (− 7.72‰ and − 1.02‰; Table 1, Fig. 8) than those of the ferroan calcite.

**Table 1:** Isotopic composition and calculated formation temperature of carbonate cements in the Zhuhai sandstones in the study area.

	Well	Depth (m)	Carbonate	δ^13^**C** (‰VPDB)	δ^18^**O** (‰VPDB)	Precipitation Temperature (℃)Precipitation Temperature (℃)		
						δ^18^O_water=-5_ (‰SMOW)	δ^18^O_water=-2_ (‰SMOW)	δ^18^O_water=0_ (‰SMOW)
1	LW4-1	1519.8	Cc	0.97	− 8.62	31	–	–
2	LW4-1	1559.8	Cc	1.58	− 9.92	38	–	–
3	LW1-1	1657.64	Cc	2.51	− 9.73	37	–	–
4	LW1-1	1657.64	Cc	1.43	− 10.44	41	–	–
5	LW1-1	1708.25	Cc	0.11	− 9.59	36	–	–
6	LW1-1	2027.25	Cc	− 0.3	− 8.28	29	–	–
7	LW1-2	2609.1	Cc	1.23	− 11.27	45	–	–
8	LW4-1	1514.8	Do	1.56	− 8.75	61	–	–
9	LW1-1	1671.45	Do	0.12	− 10.31	72	–	–
10	LW1-1	1713.5	Do	0.35	− 9.49	66	–	–
11	LW1-1	2026.5	Do	− 0.76	− 8.49	59	–	–
12	LW1-1	2037.5	Do	2.12	− 11.24	80	–	–
13	LW1-1	2037.5	Do	1.44	− 10.76	76	–	–
14	LW1-2	2577	Do	1.54	− 9.67	68	–	–
15	LW1-2	2577	Do	0.73	− 10.31	72	–	–
16	LW3-1	1861.5	Fc	− 7.48	− 13.67	60	79	94
17	LW1-1	2026.5	Fc	− 11.96	− 15.43	71	92	108
18	LW1-1	2563.5	Fc	− 24.42	− 16.63	79	101	118
19	LW1-1	2563.5	Fc	− 13.87	− 15.74	73	94	110
20	LW1-2	2609.1	Fc	− 10.72	− 14.2	63	83	98
21	LW1-2	2824.8	Fc	− 4.19	− 13.92	61	81	95
22	LW4-1	1559.8	An	− 7.53	− 14.58	88	114	137
23	LW1-1	1658	An	− 7.72	− 14.13	85	110	132
24	LW1-1	2026.5	An	− 4.48	− 12.06	71	92	109
25	LW1-1	2033.39	An	− 2.47	− 15.27	94	121	142
26	LW1-1	2045	An	− 1.02	− 17.22	111	142	167
27	LW1-2	2563.5	An	− 3.36	− 13.55	81	105	123
28	LW1-2	2701.5	An	− 2.65	− 15.46	96	123	136.25
29	LW1-2	2702.5	An	− 7.25	− 14.26	86	111	131
30	LW2-1	2997.5	An	− 6.51	− 18.05	119	152	179

### Quartz cements

**Figure 9:**
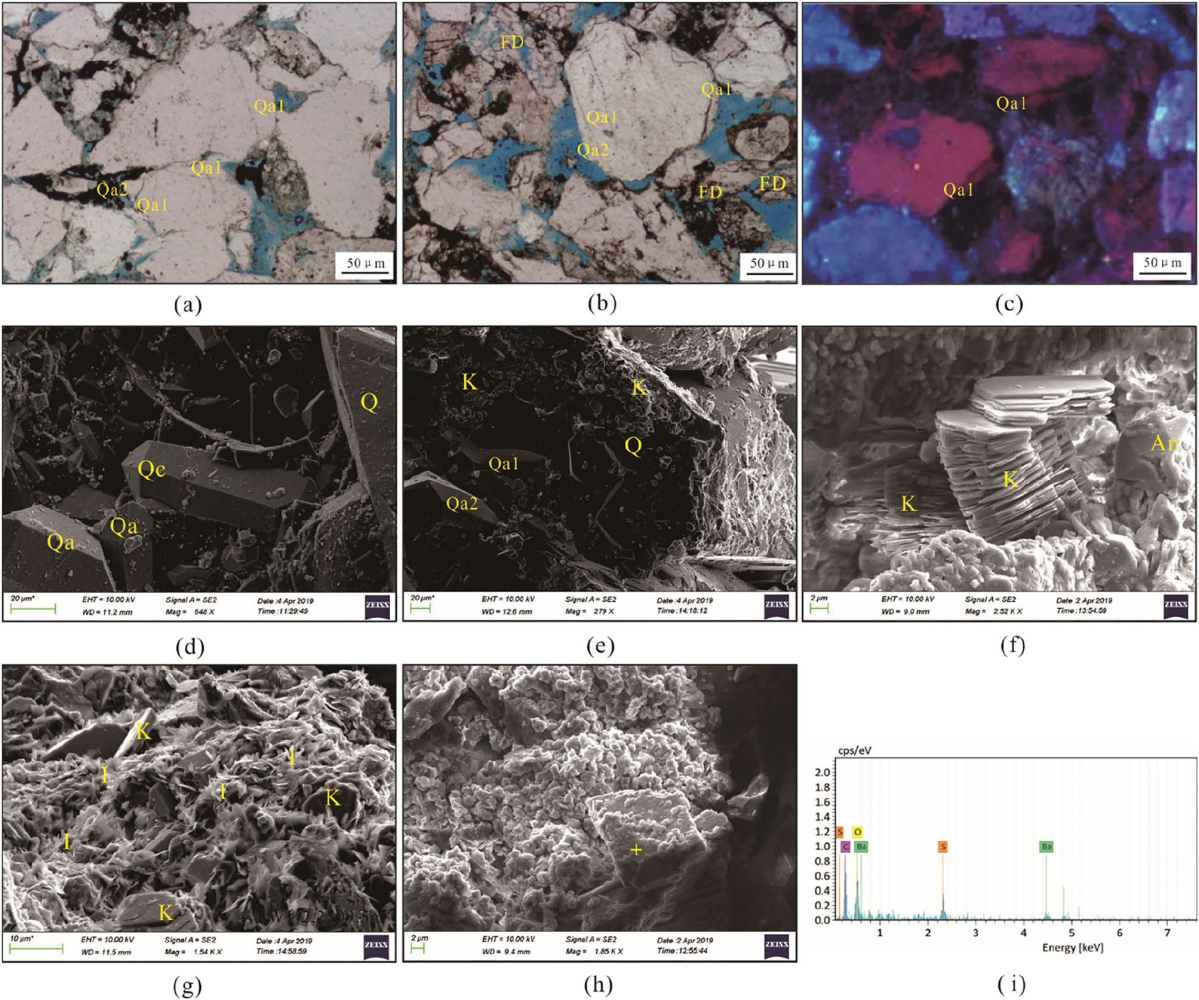
Photomicrographs of quartz, clay and other cements in the Zhuhai sandstones. (**a**) LW1-1, 1714.25 m: Two phases of quartz overgrowths; (**b**) LW1-1, 2563.5 m: Two phases of quartz overgrowths, and the dissolution of feldspar along the cleavages; (**c**) LW1-2, 2509.5 m: Syntaxial quartz overgrowths; (**d**) LW1-1, 1714.25 m: Quartz overgrowths and prismatic quartz crystals; (**e**) LW1-1, 2563.5 m: Two phases of quartz overgrowths and kaolinite cover on grain surface; (**f**) LW4-1, 1559.8 m: Kaolinite and ankerite in sandstones; (**g**) LW1-2, 2509.5 m: Transition of kaolinite to illite; (**h**) LW1-1, 2563.5 m: Barite filling among particles; and the yellow cross represents an EDS analysis point; (**i**) LW1-1, 2563.5 m: EDS-tested composition of the barite (BaSO_4_). Note: Qa1 = first phase of quartz overgrowth, Qa2 = second phase of quartz overgrowth, K = kaolinite, I = illite.

Authigenic quartz can be categorized into three types according to various morphologies: syntaxial overgrowths, small prismatic euhedral crystals, and microfracture-filling quartz cements (Fig. 9). Two stages of quartz overgrowth with thicknesses varying from 2 to 60 μm could be locally identified (Fig. 9a,b,e). The first stage of quartz overgrowth (Qa1) was enclosed or engulfed by ankerite and ferroan calcite cements (Fig. 7d), indicating that ankerite and ferroan calcite cementation occurred after Qa1. The quartz cement content is relatively low and stable, making up less than 1% (Fig. 5b), and has a positive correlation with feldspar dissolution porosity (Fig. 6c).

**Figure 10:**
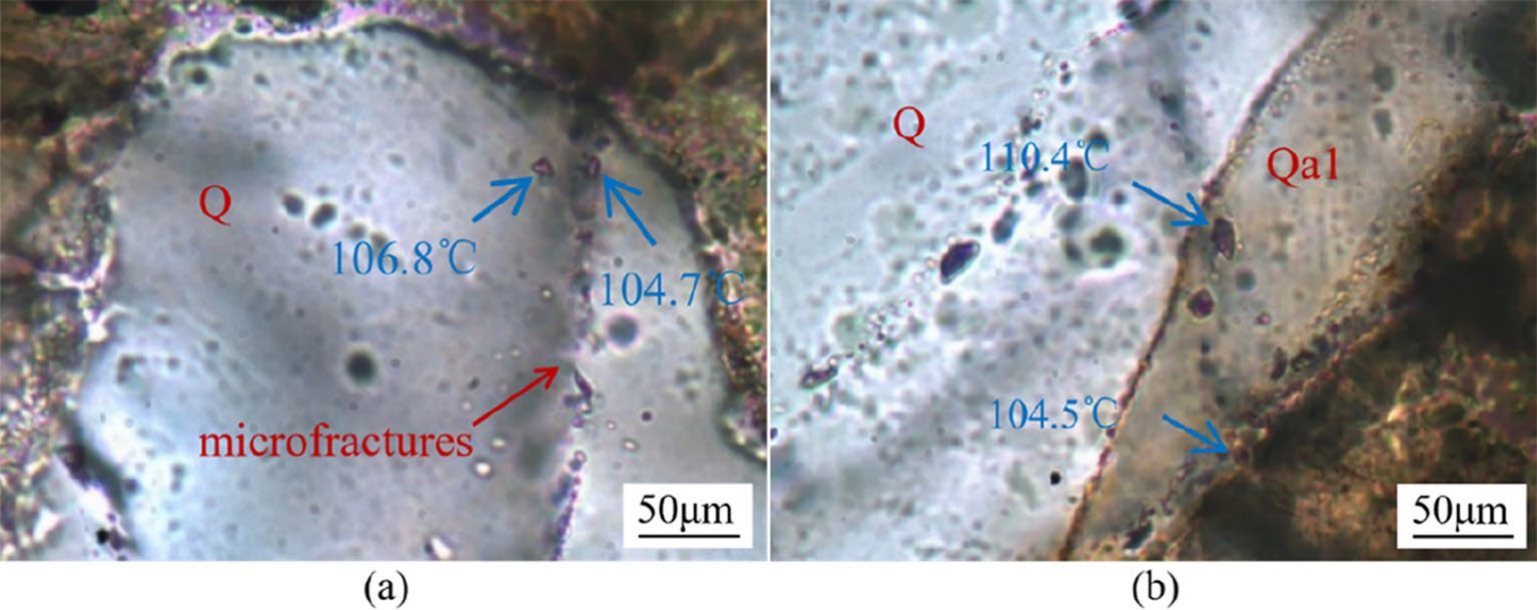
Photomicrographs of aqueous inclusions in annealed microfractures in quartz grains (**a**) and quartz overgrowths (**b**) under transmitted light observed in Zhuhai sandstones of the Baiyun Sag.

**Figure 11:**
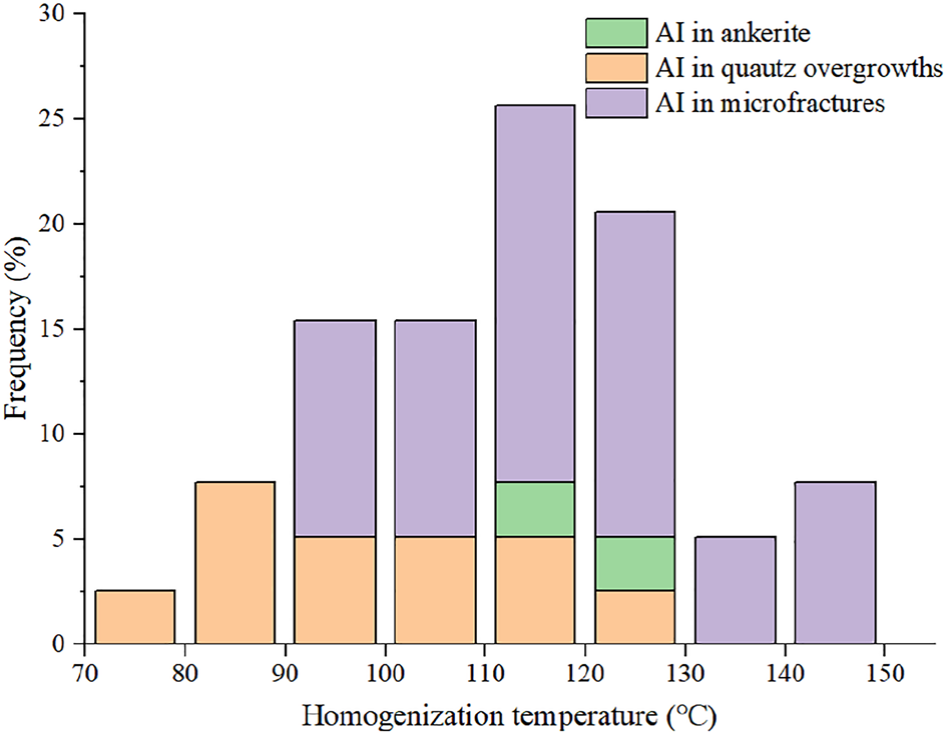
Histograms of Th for aqueous inclusions in annealed microfractures, quartz overgrowths and ankerite in Zhuhai sandstone reservoirs.

Aqueous inclusions are primarily present in the microfracture-filling quartz cements (Fig. 10a) and partly in the quartz overgrowths (Fig. 10b), with a few in carbonate cements. These aqueous inclusions are commonly two-phase liquid–vapour inclusions with a diameter range of mainly from 3 to 9.5 μm. The Th values of aqueous fluid inclusions range from 77.5 to 125 °C in the quartz overgrowths and from 94.3 to 146 °C in the microfractures (Table 2, Fig. 11). Only two Th values of aqueous fluid inclusions were measured in ankerite, 113.7 °C and 124.3 °C (Fig. 11). According to measured Th values, two stages of quartz overgrowth were confirmed. The mean Th value of aqueous inclusions in Qa1 is 89.8 °C (range 77.5–107.9 °C), and that in Qa2 is 114.3 °C (range 104.5–125 °C) (Table 2).

**Table 2:** Th of aqueous fluid inclusions in the Zhuhai sandstones of the Baiyun Sag. MF = Microfracture.

Well	Depth (m)	Inclusion location	Size (μm)	Th (℃)	Well	Depth (m)	Inclusion location	Size (μm)	Th (℃)
LW1-1	1663.54	Qa1	5.2	83.2	LW1-1	2045	MF	5.5	127
LW1-1	1663.54	Qa1	6.2	77.5	LW1-1	2045	MF	4.8	130.4
LW1-1	1663.54	Qa1	7	95.3	LW1-2	2609.1	Qa1	7.5	107.9
LW1-1	1663.54	MF	6.7	95.7	LW1-2	2609.1	MF	4	126.5
LW1-1	1663.54	MF	8	97.9	LW1-2	2609.1	MF	3.8	137.3
LW1-1	1663.54	MF	4.5	104.7	LW1-2	2609.1	MF	3	145.2
LW1-1	1663.54	MF	5.7	106.8	LW1-2	2702.5	Qa2	3.6	125
LW1-1	1708.25	Qa1	4.7	84.5	LW1-2	2702.5	Qa2	6	117.3
LW1-1	1708.25	Qa1	8.5	90.4	LW1-2	2702.5	MF	5.5	141
LW1-1	1708.25	MF	8	94.3	LW1-2	2702.5	MF	4.7	146
LW1-1	1708.25	MF	3.5	97.5	LW1-2	2702.5	MF	8.8	129
LW1-1	1708.25	MF	7	106.7	Lw3-1	1861.5	MF	4.8	112.3
LW1-1	1708.25	MF	7.5	114.8	Lw3-1	1861.5	MF	3.3	113.8
LW1-1	1708.25	MF	5	111.3	Lw3-1	1861.5	MF	9.5	117.1
LW1-1	1708.25	MF	5.5	108.2	Lw3-1	1861.5	MF	5.9	115.3
LW1-1	2045	Qa1	5.9	89.5	Lw3-1	1861.5	MF	7.1	120.1
LW1-1	2045	Qa2	5.4	104.5	Lw3-1	1861.5	MF	6.2	124.6
LW1-1	2045	Qa2	7	110.4	LW1-2	2702.5	Ank	5.5	113.7
LW1-1	2045	MF	3.3	119.3	LW1-2	2702.5	Ank	8	124.3
LW1-1	2045	MF	7.1	124.2

### Clay and other minerals

The main authigenic clay mineral types are kaolinite and illite in the studied interval, followed by illite/smectite mixed layer and chlorite. Kaolinite fills primarily primary pores as well as feldspar-dissolution pores and generally occurs in the forms of vermicular pseudohexagonal and euhedral booklet aggregates (Fig. 4b and c). The kaolinite aggregates consist mainly of thin, closely associated platelets with obvious intercrystalline microporosity (Fig. 9e and f). Some kaolinite crystals feature fibrous edges due to illitization (Fig. 9g). The honeycomb-textured illite‒smectite mixed layer features schistose crystals with a rolled border approximately 5 to 10 μm in size under SEM. Fibrous and flaky illite can also be observed in primary pores and dissolved pores and locally on grain surfaces (Fig. 9g). Similar to the quartz cement, a positive relationship also exists between the abundance of clay minerals and feldspar-dissolution porosity (Fig. 6d).

**Figure 12:**
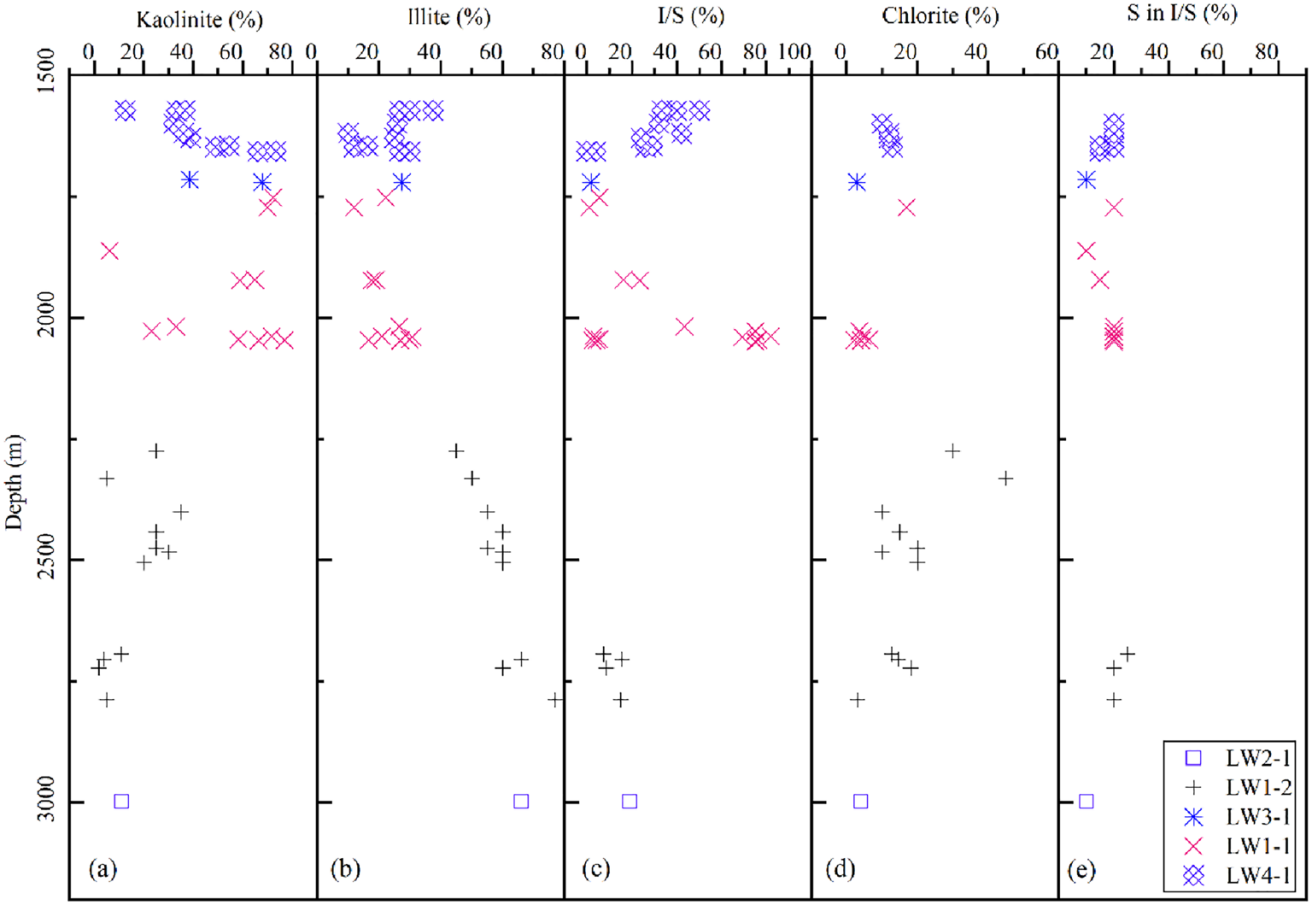
The content of clay minerals versus burial depth in the Zhuhai sandstones. X-ray diffraction data are used.

Based on XRD analysis of the clay fraction (< 2 μm) of sandstones, kaolinite is the major clay mineral and is dominant at depths above 2000 m, decreasing sharply in abundance below this depth, particularly below approximately 2500 m. Illite is dominant at depths greater than 2000 m (Fig. 12). Additionally, the contents of the illite/smectite mixed layer and chlorite have wide ranges and show marked increases from 1900 to 2100 m and from 2250 to 2350 m, respectively (Fig. 12).

Barite can be identified as a minor diagenetic mineral with small amounts of less than 1%. Based on the SEM‒EDS analysis, irregularly shaped barite (BaSO_4_) commonly fills the gaps among clastic grains in the Zhuhai sandstones (Fig. 9h and i).

## Discussion

### Burial and thermal history

**Figure 13:**
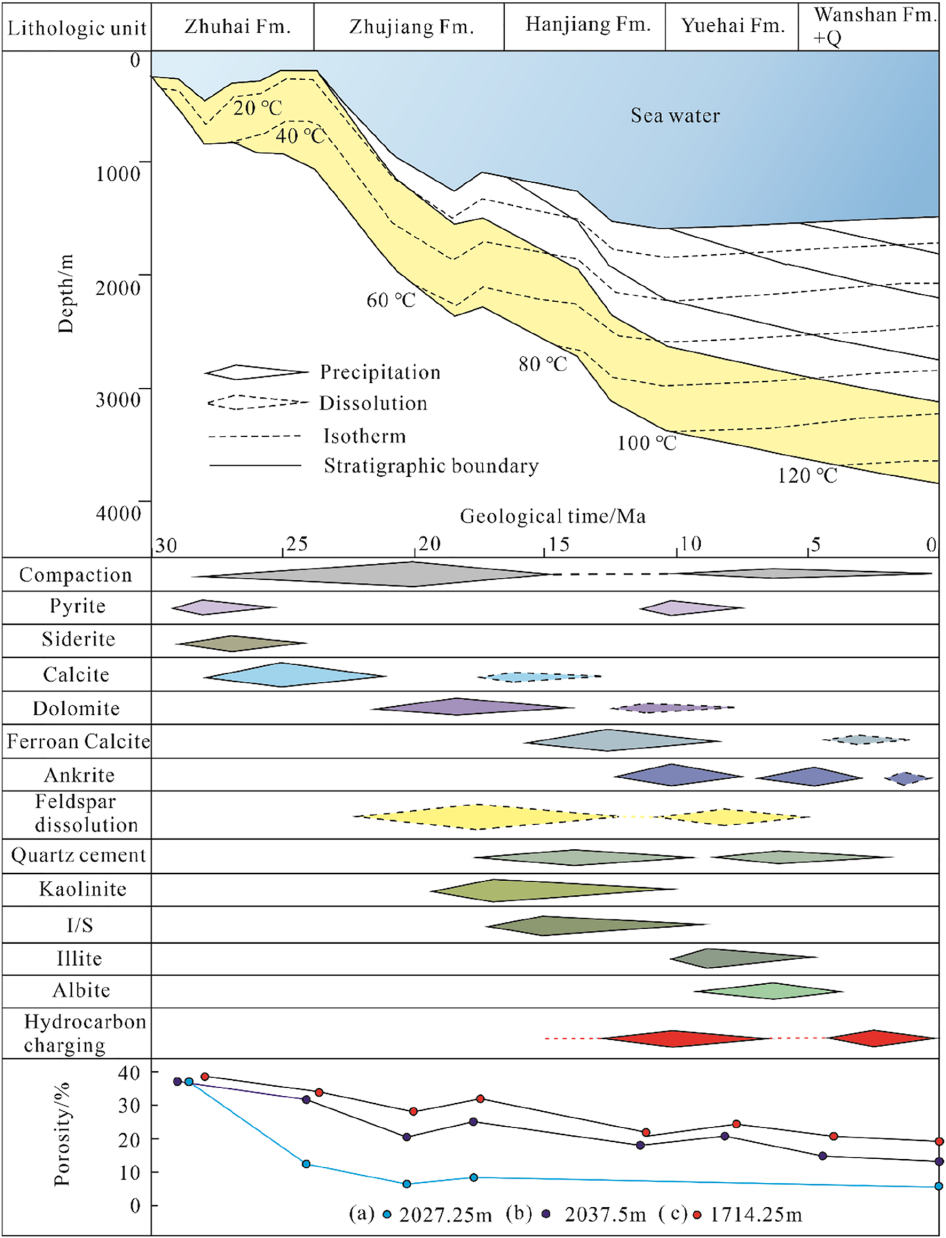
Burial history, diagenetic sequence and porosity evolution of the Zhuhai reservoirs in well LW1-1.

The Oligocene Zhuhai sequence experienced stable and persistent subsidence after deposition (Fig. 13). The overlying strata of the Zhuhai layer include up to 2200 m of deep-sea gravity flow and muddy sediments, and the average water depth at present is approximately 1450 m^27^. The Baiyun Sag is a typical hot basin, as demonstrated by previous studies, with present-day heat flow values of 24.2 to 121.0 mW/m^2^^33^. The geothermal gradient has a steady rise from northwest to southeast in the sag, which can be up to approximately 55 °C/km for the study area^34^. This discrepancy could be due to the southward transition of the spreading ridge and crustal thinning caused by the Baiyun movement since 23.8 Ma and neotectonics since ca. 13.8 Ma, along with continual deep thermal fluid upwelling in the southeastern Baiyun Sag^35^.

The average subsidence depth of the Zhuhai Formation is approximately 3600 m. The present-day maximum burial depth is approximately 3000 m, with a maximum temperature of approximately 140 °C^36^. Overpressure developed at 30 Ma and gradually increased to a peak at 13.8 Ma to 10 Ma. Afterwards, the overpressure sharply decreased and reached the present normal pressure^37^.

### Diagenetic sequence

#### Paragenetic sequence during eogenesis

A temperature of 70 °C is generally regarded as the dividing line between eogenesis and mesogenesis^8^. Compaction is the dominant eogenetic process. The precipitation of calcite and dolomite is common, especially in sandstones less than 1.0 m away from the sandstone–mudstone interface (Fig. 6b). Siderite is also demonstrated to be an eogenetic product in view of the filling relationships of these cements and primary pores between uncompacted framework grains (Fig. 7f).

According to the latitudinal gradient of the δ^18^O value of modern-day global precipitation^38^, the δ^18^O_SMOW_ values of the sedimentary water in the Baiyun sag can be considered − 5‰ (ranging from 0 to − 10‰) for the palaeolatitude of the sag at approximately 20°N. This value represents the δ^18^O_SMOW_ value of the pore fluid from which carbonate cements precipitated during eogenesis. On the basis of the fractionation equations of the oxygen isotopes for calcite–water^39^ and dolomite–water^40^, the calculated formation temperatures are between 29 and 45 °C for the calcite cements and between 59 and 80 °C for the dolomite cements (Table 2).

#### Paragenetic sequence during mesogenesis

Further compaction and feldspar dissolution were the main mesogenetic processes in the Zhuhai sandstones. The secondary pores generated from the dissolution of feldspars are observed to be mostly filled with diagenetic byproducts, such as clay and quartz cements (Figs. 4 and 5)^23,41^. The point and linear contact between detrital minerals indicates that minimal pressure dissolution has occurred in the Zhuhai sandstones. Pressure dissolution may have limited significance for quartz overgrowth^42^. The aqueous inclusions in the quartz overgrowths have a continuous Th distribution (70 °C to 130 °C) (Table 1), indicating continuous development of these cements^43^. The formation temperatures are 77.5–107.9 °C and 104.5–125°C for Qa1 and Qa2, respectively. It is inferred that feldspar dissolution and clay mineral transformation acted as an internal silica source for quartz cementation.

Petrological evidence, such as ferroan carbonate cements commonly filling feldspar-dissolution pores, suggests that these cements probably postdate the feldspar dissolution. The δ^18^O value of pore fluid becomes heavier with increasing temperature due to isotopic modification by feldspar alternation and other fluid–rock interactions^12,44^. The δ^18^O_SMOW_ values of the pore fluid during mesogenesis were assumed to be − 5‰, − 2‰, and 0‰^44^. Using a δ^18^O_SMOW_ value of − 2‰ and the fractionation equations of oxygen isotopes for calcite–water^39^ and dolomite–water^45^, the precipitation temperatures are calculated to be 79–101 °C for the ferroan calcite and 91–152 °C for the ankerite (Table 2). The measured Th of aqueous fluid inclusions in the ankerite cements (113.7 °C, 124.3 °C, Table 1) are within the results calculated for the ankerite. Therefore, it is reasonable to assume that the pore fluid had a δ^18^O_SMOW_ value of − 2‰ when the ferroan carbonate cements precipitated during mesogenesis.

Hydrocarbon inclusions are pervasively developed and are closely associated with the coeval aqueous inclusions in quartz microfractures in the Zhuhai sandstones (Fig. 10a). The Th of the coeval aqueous inclusion is regarded as the closest equivalent to the trapping temperature of the coexisting hydrocarbon inclusions, and it ranges from 94.3 to 146 °C, as previously stated. This result agrees with the previous detailed work of^46^, which showed that two periods of oil charge exist in the study area, occurring at 13.1–7.3 Ma and 5.5–0 Ma^46^. The first period of oil charge postdated the dissolution of feldspar and antedated or was synchronous with the precipitation of mesogenetic ferroan calcite. The second period of oil charge postdated the late ankerite cementation (Fig. 13).

**Figure 14:**
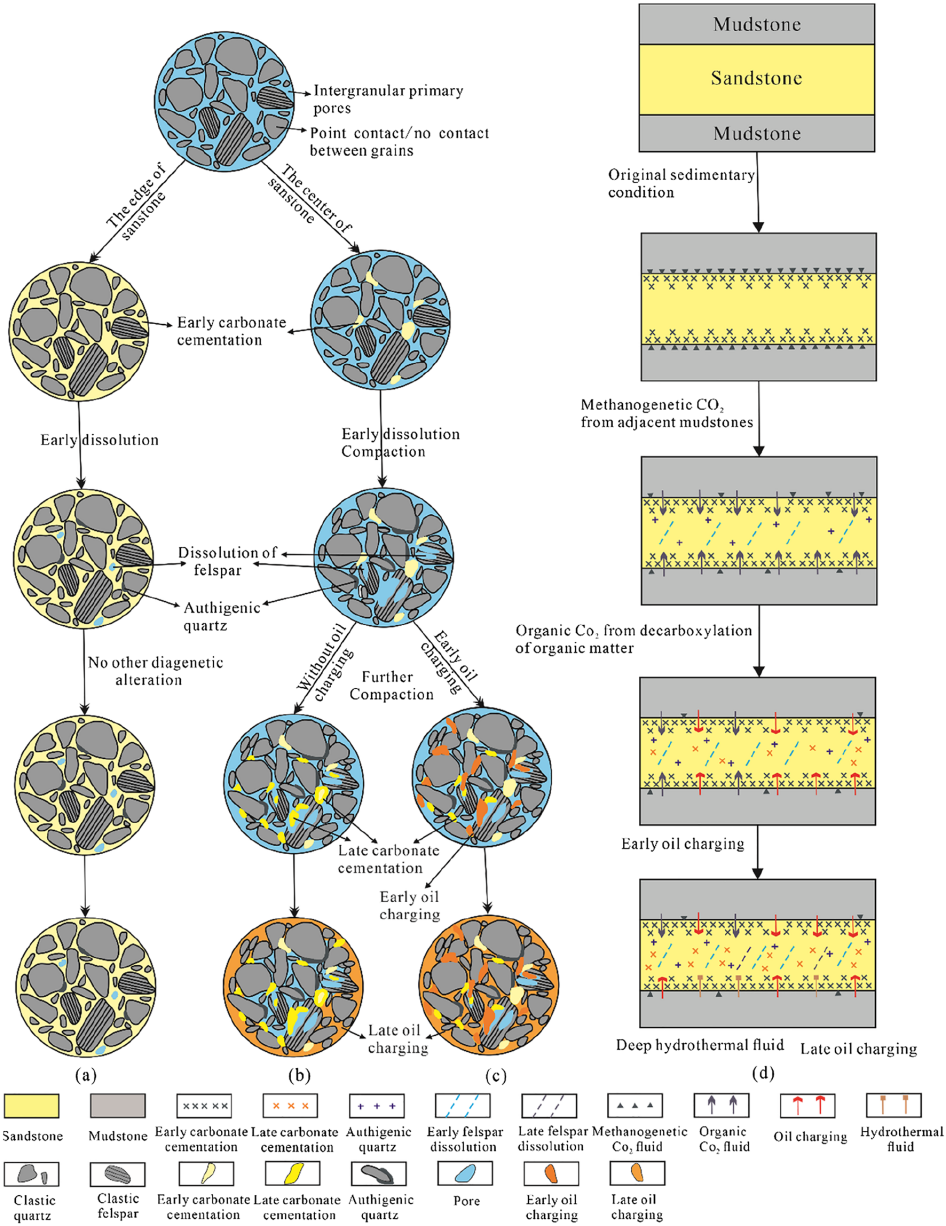
Schematic diagram showing the fluid flow and diagenetic evolution of the Zhuhai sandstones.

Figure 13 illustrates the paragenetic sequence of the main diagenetic events of the Zhuhai sandstones. However, not all reservoirs experienced the entire paragenetic sequence mentioned above^47^. Parts of reservoirs, especially thin beds or the marginal parts of thick beds (< 1.0 m), were rich in early calcite and dolomite cements during eogenesis, even filling all the intergranular spaces and becoming tight reservoirs, and hardly any other diagenetic alteration occurred (Figs. 13a and 14a). The middle parts of thick beds (> 1.0 m), by contrast, experienced complex diagenetic histories, mainly including compaction, weak early carbonate cementation, and relatively strong dissolution of feldspar (Fig. 14b,c). Subsequently, reservoirs without the early oil charge experienced strong cementation of the late carbonate, whereas the charging of late oil slowed late carbonate cementation to a certain extent (Fig. 13(b) and 14(b)). For reservoirs with the early oil charge, the selective early oil charge affected the path of diagenetic evolution; in particular, it significantly hindered late carbonate cementation. This resulted in the alteration of the wettability from water wet to oil wet. This aided the second period of oil charge (Figs. 13b and 14b).

### Evolution of pore fluid and process of fluid–rock interaction

#### Fluid flow and related fluid‒rock interactions during eogenesis

The eogenetic products record early burial fluids during the precipitation process and the climatic conditions during deposition^8,23^. The rapid subsidence of the studied interval suggests that the pore fluids during eogenesis were not significantly influenced by meteoric water. In addition, meteoric water has a δ^13^C value of − 7‰, which is much lower than the δ^13^C values of the early-formed calcite cements (− 0.76‰ to + 2.51‰), indicating that it contributed little to the precipitation of these cements.

The greater precipitation of calcite and dolomite in sandstones less than 1.0 m away from the sandstone–mudstone interface (Fig. 6b) indicates that interbedded mudstones might have been a crucial source of the early-formed carbonate cements in the sandstones. The interbedded shelf mudstones contain a considerable amount of organic matter (TOC values range from 0.66 to 1.47%, average 1.08%)^48^. The bicarbonate species related to microbial methanogenesis of organic matter (δ^13^C up to + 8‰) might have been an important carbon source for the early-formed calcite and dolomite. This is supported by the relatively positive δ^13^C values (− 0.76‰ to + 2.51‰) of these cements (Fig. 14a)^49^. In addition, the mudstones in the Zhuhai Formation are high in detrital carbonate minerals (over 15%)^50^. These minerals have δ^13^C values that vary from 0 to + 5.9‰^51^. The dissolution of the carbonate minerals in adjacent mudstones could thus be another carbon source^52^. Additionally, the Ca^2+^ and Mg^2+^ ions dissolved in the mudstone were probably transported to sandstone as well.

During this stage, pore fluids from the mudstones interbedded with the sandstones were mainly driven by compaction (Fig. 14d). Large-scale mass transfer occurred from the mudstones to adjacent sandstones, implying that a relatively open diagenetic environment occurred within a local range (e.g., several metres). The rapid subsidence and extensive mechanical compaction of the mudstones during eogenesis may have expelled fluids into adjacent sandstone reservoirs^53^. However, much of the research to date has demonstrated that the rate of fluid flow from mudstones to sandstones is rather low, only several millimetres per year^4,54^. Thus, advection and convection likely made an insignificant contribution to mass transport. With the dissolution of carbonate minerals, microbial alteration of organic matter in mudstones alters the ion concentration of pore fluids, creating steep diffusion gradients. The diffusive transport of dissolved ions from mudstones to sandstones may have had a great impact on the distribution pattern of the early carbonate cements. Similar conclusions have been reached in other sedimentary basins^8,54,55^.

#### Fluid flow and related fluid–rock interactions during mesogenesis

Due to thermal maturation of kerogen (temperature > 70 °C), a chemical gradient was formed between the source rock and adjacent sandstones. Organic CO_2_ and acids were transported via diffusion. This resulted in a certain amount of feldspar dissolution^56,57^. However, the dissolution of feldspar barely occurred near the edge but rather occurred in the middle part of the sandbodies (Fig. 6). The most likely cause is strong carbonate cementation near the sandstone–mudstone interface during diagenesis, resulting in tight layers forming along the sandbody edges, which control the transport of pore fluids rich in organic CO_2_ and acids, crossing the sandbody edge and reaching the porous zone in the centre of the sandbody (Fig. 14b).

The solubility of silica is extremely low^42,54^. Together with the relatively limited flux of formation water, which is difficult over long distances and in large quantities, the distribution patterns of quartz cement indicate little or no external silica involvement (Fig. 6)^54^. The intimate association between diagenetic mineral assemblages (Fig. 6) suggests that the silica source of quartz cement is closely related to feldspar dissolution. This is also supported by the precipitation temperature zone of Qa1 (77.5 °C to 107.9 °C, average 89.8 °C). Thus, the dissolution of feldspar may have served as an important silica source for Qa1^34,42,58^. As mentioned before, kaolinite is mainly stable above 2000 m (temperature < 100 °C), and the rapid and mass illitization of kaolinite occurs in the sandstone below 2000 m, especially between 2200 and 2500 m (with temperatures from 100 to 120 °C). The formation temperature of Qa2 ranges from 104.5 to 125 °C, with an average of 114.3 °C, coinciding with the optimal temperature zone of the illitization of kaolinite. It seems highly likely that the illitization of kaolinite was a major source of silica for Qa2 in the study area.

The relatively negative δ^13^C values (− 24‰ to − 1‰) of the ferroan calcite suggest an organic source from adjacent mudstones.

(δ^13^C_VPDB_ from − 25 to − 10‰) (Table 2)^49^. In addition, the precipitation of ferroan carbonate replacing nonferroan carbonate can be caused by abundant organic CO_2_ that can dissolve some early-formed carbonate. Thus, the ferroan carbonate precipitation during early mesogenesis was dominated by a mixture of carbon sources from the decarboxylation of organic matter and dissolution of early-formed carbonate cements. The illitization of smectite (60–100 °C) in the mudstones is consistent with the temperature range of the ferroan carbonate cements^25^, which can release Fe^2+^ that is transported to sandstones via diffusion, and this process may have served as the main source of the ferroan carbonate cements in the sandstone^49,59,60^, as evidenced by the distribution pattern of carbonate cements proximal and distal to the sandstone–mudstone interface. It is inferred that there was a relatively open system for organic CO_2_ and Fe ^2+^ ion transfer on the local scale during early mesogenesis, and the predominant dissolved material was driven by diffusive transport.

The ankerite cements have higher δ^13^C values than the ferroan calcite and formed at significantly higher temperatures (91–152 °C), suggesting another origin from CO_2_-containing deep hydrothermal fluid (Fig. 14d). The hydrothermal fluid from depth had δ^13^C values of approximately − 5 ± 2‰ and generally positive δ^18^O values ranging from + 5.0 to + 7.0‰^61^. The increasing δ^13^C and δ^18^O values of the ankerite with increasing temperature may be related to the ascent and intrusion of hydrothermal fluid from depth to the sandstones. This conclusion is supported by the precipitation of barite and albite, which are typical hydrothermal minerals, as noted by others^28,29,62^. It is likely that diffusion was predominant in a locally open system with migration of hydrothermal fluid during late mesogenesis. Further study with more focus on this hydrothermal fluid is suggested.

**Figure 15:**
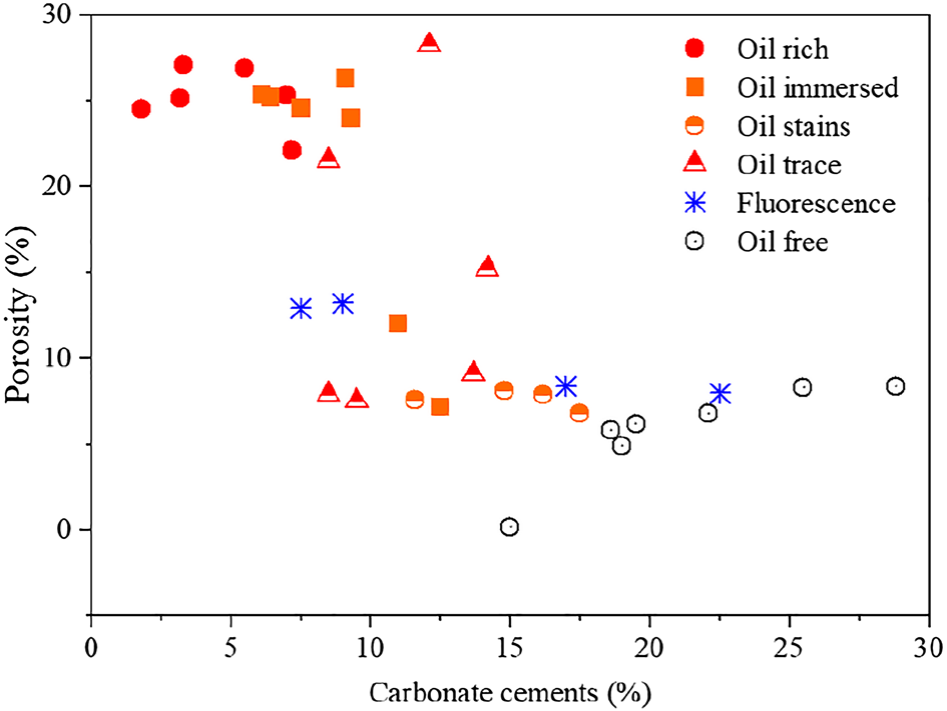
Binary diagram of porosity versus carbonate cement content in sandstones with different oil contents.

Oil charge may inhibit the compaction and late carbonate cementation of reservoirs, consequently acting to preserve porosity^63,64^. In the studied interval, the oil saturation of the reservoirs steadily increases as the content of carbonate cements decreases, which corresponds to a gradual increase in porosity (Fig. 15). There are numerous differences between the two periods of oil charge in terms of the impact on reservoir quality^64^. The first period of oil charging effectively inhibited the precipitation of ferroan calcite and ankerite prior to mesogenetic cementation (Fig. 14b,c). The first period of oil charge was synchronous with or subsequent to the transformation of feldspar to kaolinite. Both of the events may have altered the reservoir wettability to oil wet, which helped the subsequent oil charging and entrapment^64–66^. Overall, high-quality reservoirs might be present in the middle part of the thickly bedded delta sandstones, and these reservoirs experienced both periods of oil charging.

## Conclusions

This paper presents the evolution of fluids with progressive burial and the complex interactions with sandstone reservoirs, as exemplified by the Oligocene Zhuhai sandstones in the Baiyun Sag. On the basis of detailed analyses of petrographic, mineralogical, and geochemical features in the Zhuhai sandstones, this study has demonstrated the following.

(1) The Oligocene Zhuhai sandstones experienced compaction and calcite and dolomite cementation in the eogenetic stage. Subsequently, they underwent two phases of feldspar dissolution and concomitant quartz overgrowth formation, the cementation of ferroan calcite and ankerite, the conversion of clay minerals, and two periods of oil charging in the mesogenetic stage. The precipitation of typical hydrothermal minerals (such as authigenic albite and barite) was synchronous with ankerite in the late mesogenetic stage.

(2) The pore fluids were influenced by microbial methanogenesis and carbonate mineral dissolution in adjacent mudstones during the eogenetic stage. Large-scale mass transfer occurred along steep diffusion gradients in a relatively open geochemical system and resulted in abundant precipitation of calcite or dolomite within 1.0 m of the sandstone–mudstone interface. This conclusion is supported by the heavier carbon and oxygen isotopic compositions during eogenesis.

(3) Thermal maturation of organic matter significantly controlled the pore fluids in the early mesogenetic stage. Organic acid and CO_2_ contributed to the dissolution of feldspar and early cements. Diffusion-migrated dissolved material was mainly reprecipitated in situ or in adjacent pores in the form of authigenic quartz and ferroan calcite. This conclusion is supported by the obviously lighter isotopic compositions of the early mesogenetic ferroan calcite. During late mesogenesis, dissolution might have been partly driven by deep hydrothermal fluid. Evidence that supports this interpretation includes the precipitation of typical hydrothermal minerals and the abnormally high isotopic compositions of the late ankerite.

(4) Oil charging, especially the first period of oil charging, may have inhibited carbonate cementation and compaction, accordingly causing the preservation of porosity. This could have altered the reservoir wettability from water wet to oil wet in combination with the precipitation of authigenic kaolinite caused by feldspar dissolution. The multistage pore-fluid activity and related fluid–rock interaction lead to the potential for high-quality reservoirs developed in the middle part of the thickly bedded delta sandstones that experienced both periods of oil charge.

## Data Availability

All data generated or analysed during this study are included in this published article.
